# A drug–drug interaction study to assess the potential effect of acid-reducing agent, lansoprazole, on quizartinib pharmacokinetics

**DOI:** 10.1007/s00280-019-03915-1

**Published:** 2019-08-05

**Authors:** Jianke Li, Denise Trone, Jeanne Mendell, Patrick O’Donnell, Natalie Cook

**Affiliations:** 1 Daiichi Sankyo, Inc., 10201 Wateridge Circle, Suite 240, San Diego, CA 92121 USA; 2 Daiichi Sankyo, Inc., Basking Ridge, NJ USA

**Keywords:** Quizartinib, Proton pump inhibitor, Gastric acid modifier, Drug interaction, AML

## Abstract

**Purpose:**

Quizartinib, a potent, selective FMS-like tyrosine kinase 3 (*FLT3*) inhibitor, is currently in phase 3 development for patients with *FLT3*–internal tandem duplication-mutated acute myeloid leukemia (AML). Acid-reducing agents (ARAs; e.g., proton pump inhibitors) are frequently used during AML treatment. Since quizartinib demonstrates pH-dependent solubility, the effect of lansoprazole coadministration on pharmacokinetics (PK) of quizartinib tablet formulation was assessed.

**Methods:**

An open-label, parallel-group study randomized 64 healthy adults to single-dose quizartinib 30 mg alone (reference) or lansoprazole (60 mg once daily, days 1–5) + single-dose quizartinib 30 mg (day 5) (test). Plasma concentrations of quizartinib and its active metabolite, AC886, were measured to 504 h postdose; the effect of lansoprazole on quizartinib PK was assessed by analysis of variance.

**Results:**

Quizartinib geometric mean ratios (test/reference) and 90% confidence intervals for maximum observed plasma concentration (*C*_max_), area under the concentration–time curve to last measurable drug concentration (AUC_last_), and AUC to infinity were 86.11% (78.4%, 94.6%), 93.96% (79.6%, 110.9%), and 95.30% (80.2%, 113.3%), respectively. Comparisons showed a modest decrease in quizartinib absorption when co-administered with lansoprazole, with lower limits for *C*_max_ and AUC_last_ just below 80–125% limits. Treatment-emergent adverse events were mild or moderate; the most frequent in either treatment group were headache [quizartinib alone: (*n* = 3) 10%], upper respiratory tract infection [quizartinib alone: (*n* = 2) 6.7%; lansoprazole + quizartinib: (*n* = 3) 9.1%], and muscle tightness [quizartinib alone: (*n* = 2) 6.7%].

**Conclusions:**

Concomitant lansoprazole had minimal effect on quizartinib PK as a formulated tablet, indicating that quizartinib can be administered with ARAs.

**Electronic supplementary material:**

The online version of this article (10.1007/s00280-019-03915-1) contains supplementary material, which is available to authorized users.

## Introduction

The majority of patients with acute myeloid leukemia (AML) have poor prognosis despite recent advances in therapy because of disease heterogeneity driven by clonal evolution, which contributes to treatment resistance [1–3]. Patients with mutations in the internal tandem duplication (ITD) region of the FMS-like tyrosine kinase 3 (*FLT3*) gene, one of the most common driver mutations in AML, have particularly poor prognosis following conventional chemotherapy [1, 2, 4]. Quizartinib is an orally administered, highly potent and selective next-generation *FLT3* inhibitor currently in phase 3 development in patients with *FLT3*–ITD-mutated AML (QuANTUM-R: NCT02039726; QuANTUM-First: NCT02668653). Quizartinib treatment in previous phase 2 trials demonstrated composite complete remission (CRc) rates of 44–47% in patients with relapsed or refractory *FLT3*–ITD-mutated AML, and 34–42% of patients were bridged to potentially curative hematopoietic stem cell transplant [5–7]. Quizartinib was generally well tolerated; the main adverse events (AEs) were manageable myelosuppression and QT prolongation that was mitigated with dose reduction [5, 7, 8]. Results from the completed QuANTUM-R phase 3 study in patients with relapsed/refractory *FLT3*–ITD-mutated AML, quizartinib (30-mg starting dose with escalation to 60 mg once daily; *n* = 245) showed prolonged overall survival versus standard chemotherapy (*n* = 122; median 6.2 months vs. 4.7 months, respectively; hazard ratio: 0.76; *P *= 0.02), with a greater proportion of patients undergoing hematopoietic stem cell transplantation (32% vs. 11%, respectively) [9].

Patients with AML often experience gastrointestinal AEs (i.e., diarrhea, nausea, and vomiting) with antileukemia treatment [10, 11], including quizartinib [5, 7, 12]. Acid-reducing agents (ARAs), such as proton pump inhibitors (PPIs), may be administered as part of supportive care to patients with AML [13, 14]. In one assessment, approximately 20–33% of cancer patients received ARAs, of which PPIs were the most commonly prescribed [15]. PPIs increase intragastric pH and may subsequently reduce the solubility and bioavailability of oral tyrosine kinase inhibitors (TKIs) [16].

In vitro data indicate that quizartinib dihydrochloride has pH-dependent solubility. It is important to understand factors influencing pharmacokinetics (PK) to ensure appropriate exposure to oral TKIs such as quizartinib. Previous PK studies in healthy subjects and patients with AML [12, 17] indicated that quizartinib reaches maximum concentrations at 4 h (range 2–8 h) and demonstrates dose-proportional PK at doses ranging from 20 to 90 mg, with an accumulation ratio of approximately 4 (data on file, Daiichi Sankyo Inc., Basking Ridge, NJ, USA). Quizartinib is metabolized to its pharmacologically active primary metabolite AC886, which has similar potency and selectivity as the parent [dissociation constant (Kd) values for *FLT3* of 1.3 nM for quizartinib and 0.54 nM for AC886], with a metabolite–parent ratio of 60% following repeated daily dosing in patients with AML (data on file, Daiichi Sankyo Inc., Basking Ridge, NJ, USA). Quizartinib and AC886 half-lives were estimated as 73 h and 119 h, respectively, in AML patients (data on file, Daiichi Sankyo Inc., Basking Ridge, NJ, USA).

We report results from a phase 1 study that examined the effects of lansoprazole, a PPI, on the PK of quizartinib (in tablet formulation) and its active metabolite AC866.

## Methods

### Study design

This phase 1, open-label, randomized, parallel-group study was conducted at a single center in the USA from January to May 2015 to determine the effect of gastric pH modification by lansoprazole on the PK of quizartinib and its active metabolite, AC886, in healthy subjects. The tolerability and safety of quizartinib administered alone and in combination with lansoprazole were also assessed. The study was conducted in compliance with the Declaration of Helsinki and the International Conference on Harmonisation (ICH)/Good Clinical Practice as well as all applicable state, local, and federal regulations. The study protocol, amendments, and informed consent forms were reviewed and approved by the institutional review board. All subjects provided written informed consent before any study-related procedure took place.

### Eligibility

Healthy males and nonpregnant females, 18–55 years of age, were eligible. Key inclusion criteria were body mass index of 18–32 kg/m^2^; serum potassium, magnesium, and calcium within normal limits; adequate hepatic and coagulation parameters; and adequate renal function, as defined by serum creatinine ≤ 1.5 × upper limit of normal and estimated creatinine clearance at screening ≥ 80 mL/min according to the Cockcroft–Gault equation. Main exclusion criteria were history of clinically significant disease, abnormality, or drug allergy; treatment with any investigational product in a clinical study within 30 days (or five drug-half-lives, whichever was longer); and use or anticipated use of prescription medications including hormonal contraceptives, over-the-counter medications, herbal products, or dietary supplements. Subjects who received drugs with a risk of QT prolongation or torsade de pointes within 14 days of study initiation were excluded.

### Randomization and treatments

Subjects were randomized into two groups: lansoprazole and quizartinib (lansoprazole + quizartinib; test group) and quizartinib alone (reference group). On days 1–5, subjects in the test group were administered 60-mg lansoprazole (2 × 30 mg oral delayed-released capsule) once daily in the morning before food. On day 5, subjects in both groups received quizartinib as a single dose in tablet form containing 30-mg quizartinib dihydrochloride (equivalent to 26.5-mg quizartinib free-base) after a 10-h fast and continued fasting for at least 4 h after dosing. All study drugs were taken with approximately 240 mL of water. Test group subjects received the 60-mg lansoprazole dose concomitantly with quizartinib on day 5.

The study drug doses were selected because they were considered appropriate to assess a maximum potential drug–drug interaction between quizartinib and lansoprazole based on the starting dose for quizartinib in the pivotal phase 3 study (QuANTUM-R, NCT02039726), and the highest daily lansoprazole dose of 60 mg received by AML patients in a quizartinib phase 2 study (NCT00989261). For this study, 30 mg was selected due to safety considerations in healthy volunteers, and 30 mg was the highest dose strength for the tablet formulation.

### Sample collection and pharmacokinetic analyses

Blood samples for measurement of plasma quizartinib and AC886 concentrations were collected from all subjects before quizartinib dosing on day 5 and at 0.25, 0.5, 1, 2, 3, 4, 5, 6, 8, 12, 24, 36, 48, 72, 96, 120, 144, 168, 192, 216, 288, 360, 432, and 504 h after quizartinib administration. Plasma concentrations were determined by BASi (West Lafayette, IN, USA) using a validated analytical method.

The PK population consisted of all subjects who received the quizartinib dose and had evaluable maximum observed plasma concentration (*C*_max_) or area under the plasma concentration–time curve from time 0 to the last quantifiable plasma concentration (AUC_last_) or from time 0 extrapolated to infinity (AUC_inf_) for quizartinib, AC886, or quizartinib + AC886, and did not have any significant protocol deviations. PK parameters in plasma for quizartinib, AC886, and quizartinib + AC886, including *C*_max_, AUC_last_, AUC_inf_, terminal elimination half-life (*T*_1/2_), time to *C*_max_ (*T*_max_), apparent systemic clearance (CL/*F*), and apparent volume of distribution (*V*_z_/*F*), were calculated using WinNonlin^®^ version 6.4 (Certara USA, Inc., Princeton, NJ, USA). In PK analyses of quizartinib and AC886, concentration values that were below the limit of quantification (BLQ) were set to zero unless flanked by measurable concentrations, in which case they were set to missing. In PK analyses of quizartinib + AC886, concentration values that were BLQ for both quizartinib and AC886 were set to zero. If AUC_inf_ could not be determined for quizartinib and AC886, then AUC_inf_ was not reported for quizartinib + AC886.

### Bioanalytic methods

Quizartinib and AC886 plasma concentrations were measured by BASi (Bioanalytical Systems Inc., West Lafayette, IN, USA) using a validated liquid chromatography (LC)–tandem mass spectrometry (MS) method. Quizartinib and AC886 were extracted from K_2_EDTA human plasma by solid-phase-supported liquid extraction with methyl tert-butyl ether; d_4_-quizartinib and d_4_-AC886 were added as internal standards prior to extraction. Samples were injected into an LC–MS/MS system (LC: Nexera System, Shimadzu Scientific Instruments, Columbia, MD, USA; MS: API 5500™, SCIEX, Framingham, MA, USA) using a PFP column (Phenomenex/Kinetex, Torrance, CA, USA) with a gradient ammonium formate/formic acid/acetonitrile/water mobile phase. The monitored mass-to-charge (m/z) values for the precursor ions were 561.1, 577.1, 565.1, and 581.1 for quizartinib, AC886, d_4_-quizartinib, and d_4_-AC886, respectively. The m/z values monitored for the product ions were 421.1 for quizartinib and AC886, and 425.1 for d_4_-quizartinib and d_4_-AC886. For both quizartinib and AC886, the analytical range validated was from 0.50 to 500 ng/mL. For quizartinib at the lower limit of quantitation (LLOQ), precision was 5.2% coefficient of variation (CV) and accuracy was 1.0% bias across 16 runs; accuracy ranged from −10.2 to 7.8% bias within runs. For quizartinib at the upper limit of quantitation, precision was 3.5% CV and accuracy was −1.6% bias across 16 runs; accuracy ranged from −6.6 to 6.4% bias within runs. For AC886 at the LLOQ, precision was 6.0% CV and accuracy was 0.2% bias across 14 runs; accuracy ranged from −7.9 to 10.6% bias within runs. For AC886 at the upper limit of quantitation, precision was 2.9% CV and accuracy was −1.4% bias across 14 runs; accuracy ranged from −6.4 to 4.6% bias within runs.

### Safety analysis

The safety analysis population consisted of all randomized subjects who received quizartinib or at least one dose of lansoprazole. Safety was assessed with physical examinations, vital signs, AE evaluations, 12-lead electrocardiograms (ECGs), and clinical laboratory tests (hematology, chemistry, and urinalysis). AEs were evaluated during the study according to the Medical Dictionary for Regulatory Activities version 17.1 and assessed for severity and relation to study drugs. Laboratory results were summarized based on National Cancer Institute Common Terminology Criteria for Adverse Events version 4.03.

As quizartinib was associated with QTcF (QT interval corrected with Fridericia’s formulation) prolongation in an earlier study [12], ECGs were performed on all subjects at screening and on days −1 and 5. On day 5, ECGs were obtained before quizartinib administration and at 2, 3, 4, and 8 h after quizartinib administration. Analyses of QTcF interval data were based on ICH E14 categories [18].

### Statistics

A sample size of 25 subjects per group was determined to yield a ≤ 10% relative standard error of the mean, based on the observed intersubject coefficient of variation (CV%) of approximately 60% for PK parameters in a previous drug–drug interaction study in healthy volunteers (data on file, Daiichi Sankyo, Inc., Basking Ridge, NJ, USA). Up to 32 subjects/group were planned to account for possible premature withdrawal. Descriptive statistics were used to summarize plasma concentrations and PK parameters of the PK analysis population. Safety parameters were summarized in the safety analysis population using descriptive statistics. All descriptive statistics were calculated using SAS^®^ software version 9.3 (SAS Institute Inc., Cary, NC, USA). The effects of lansoprazole on PK of quizartinib, AC886, and quizartinib + AC886 were assessed by comparing *C*_max_, AUC_inf_, and AUC_last_ of the two treatment arms using an analysis of variance (ANOVA) model with treatment as a fixed effect. PK parameters were natural logarithm (ln)-transformed prior to analyses. The ln-transformed PK results were back-transformed by exponentiation to obtain the geometric least squares mean (LSM) for each treatment and calculate geometric LSM ratios for pairwise comparisons. Absence of a drug–drug interaction was concluded if the 90% confidence intervals (CIs) of geometric LSM ratios (test group:reference group) for *C*_max_ and AUC were completely contained within the accepted 80–125% interval, in accordance with regulatory agency guidelines [19, 20].

## Results

### Demographics and baseline characteristics

A total of 64 subjects were enrolled, with 31 randomized to the quizartinib-alone group and 33 to the lansoprazole + quizartinib group (Online Resource 1). Overall, five subjects did not complete the study. One subject from the quizartinib-alone group was withdrawn from the study prior to dosing because of a low platelet count and was excluded from the safety population; four subjects from the lansoprazole + quizartinib group withdrew consent. Demographic and baseline characteristics of the safety population were generally similar between treatment groups (Table 1). The groups were balanced with respect to age, sex, weight, and body mass index. The racial composition differed somewhat between groups, with the test group having a lower percentage of white subjects (54.5% vs. 66.7%) and a higher percentage of black (30.3% vs. 23.3%) and “other” (15.2% vs. 6.7%) subjects.

**Table 1:** Demographics and baseline characteristics of subjects in the study

	Quizartinib, *n* = 30^a^	Lansoprazole + quizartinib, *n* = 33	Overall, *n* = 63
Age, years, mean (SD)	36.4 (9.1)	32.9 (9.0)	34.6 (9.1)
Sex, *n* (%)			
Female	13 (43.3)	14 (42.4)	27 (42.9)
Male	17 (56.7)	19 (57.6)	36 (57.1)
Race, *n* (%)			
White	20 (66.7)	18 (54.5)	38 (60.3)
Black	7 (23.3)	10 (30.3)	17 (27.0)
Asian	1 (3.3)	0	1 (1.6)
Other	2 (6.7)	5 (15.2)	7 (11.1)
Weight, kg, mean (SD)	74.7 (10.0)	75.7 (11.4)	75.2 (10.7)
Body mass index, kg/m^2^, mean (SD)	25.9 (3.2)	25.8 (2.8)	25.8 (3.0)

*SD* standard deviation

^a^One subject in the quizartinib-alone group was withdrawn due to low platelet count on day 4 before receiving quizartinib and was not included

### Pharmacokinetic results

**Fig. 1:**
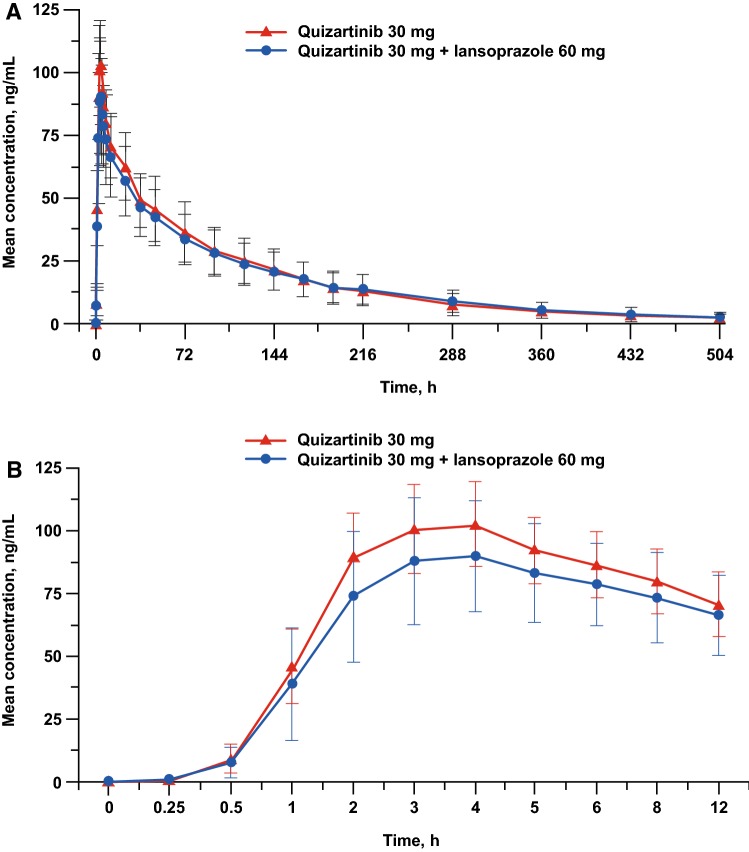
Mean (± standard deviation) concentration–time profiles of quizartinib in plasma after administration of a single 30-mg dose of quizartinib alone or with 2 × 30 mg lansoprazole (linear scale) from time zero to 504 h (**a**) and from time zero to 12 h (**b**)

The mean plasma concentration–time profiles of quizartinib were similar between the two treatment groups (Fig. 1a, b). Plasma PK parameters for quizartinib are summarized in Table 2. Median *T*_max_ was 3.5 h with lansoprazole + quizartinib and 4.0 h with quizartinib alone. Geometric mean *C*_max_ values were slightly lower after coadministration of lansoprazole + quizartinib than after quizartinib alone (90.3 vs. 105 ng/mL, respectively); *C*_max_ values demonstrated low variability for both treatments, with CV of 27.2% and 16.3%, respectively. Similarly, geometric mean AUC_last_ and AUC_inf_ were also slightly lower for lansoprazole + quizartinib than for quizartinib alone (AUC_last_: 7830 vs. 8330 ng h/mL; AUC_inf_: 8260 vs. 8660 ng h/mL, respectively); AUC values demonstrated medium variability in both groups (CV 45.0–46.4% with lansoprazole + quizartinib; CV 35.2–37.4% with quizartinib alone). Geometric mean CL/*F* values were comparable between treatment groups (3.2 L/h with lansoprazole + quizartinib and 3.1 L/h with quizartinib alone); geometric mean *V*_z_/*F* values for quizartinib were slightly higher for lansoprazole + quizartinib compared with quizartinib alone (479 and 431 L, respectively). Geometric mean *T*_1/2_ was comparable between groups (107.4 h for lansoprazole + quizartinib and 102.2 h for quizartinib alone).

**Table 2:** Plasma PK parameters of quizartinib administered with and without lansoprazole

PK parameter	Quizartinib, *n* = 30	Lansoprazole + quizartinib, *n* = 32
Median *T*_max_ (min, max), h	4.0 (2.0, 8.0)	3.5 (2.0, 8.0)
*C*_max_ (CV%), ng/mL^a^	105 (16.3)	90.3 (27.2)
AUC_last_ (CV%), ng h/mL^a^	8330 (35.2)	7830 (45.0)
AUC_inf_ (CV%), ng h/mL^a^	8660 (37.4)	8260 (46.4)^b^
Mean *T*_1/2_ (SD), h^c^	102.2 (29.0)	107.4 (28.0)^b^
CL/*F* (CV%), L/h^a^	3.1 (37.4)	3.2 (46.4)^b^
*V*_z_/*F* (CV%), L^a^	431 (28.4)	479 (39.0)^b^

*AUC*_*inf*_ area under the concentration–time curve from time 0 to infinity, *AUC*_*last*_ area under the concentration–time curve from time 0 to the time of the last quantifiable concentration, *CL/F* apparent clearance, *C*_*max*_ maximum observed concentration, *CV* coefficient of variation, *PK* pharmacokinetic, *SD* standard deviation, *T*_*1/2*_ apparent terminal phase elimination half-life, *T*_*max*_ actual sampling time to reach maximum observed concentration, *V*_*z*_*/F* apparent volume of distribution in the terminal phase

^a^Geometric mean

^b^*n* = 31

^c^Arithmetic mean (SD)

### Effect of lansoprazole on quizartinib PK

Quizartinib exposure decreased slightly after repeated lansoprazole administration. Quizartinib *C*_max_, AUC_last_, and AUC_inf_ were 13.9%, 6.0%, and 4.7% lower, respectively, for lansoprazole + quizartinib than for quizartinib alone (Table 3). The bounds of the 90% CI for *C*_max_ (78.4%, 94.6%) and AUC_last_ (79.6%, 110.9%) were slightly outside of the accepted 80–125% interval for establishing an absence of a significant drug–drug interaction, while the 90% CI for AUC_inf_ (80.2%, 113.3%) fell within the accepted range.

**Table 3:** Statistical comparisons (ANOVA) of quizartinib PK parameters after a single 30-mg dose of quizartinib alone or with lansoprazole

PK parameter	Quizartinib	Lansoprazole + quizartinib	Ratio of geometric LS mean, %^a^	90% CI for ratio of geometric LS mean, %
	Geometric LS mean, *n* = 30	Geometric LS mean, *n* = 32		
*C*_max_, ng/mL	104.8	90.3	86.11	78.36, 94.64
AUC_last_, ng h/mL	8328.9	7825.6	93.96	79.63, 110.86
AUC_inf_, ng h/mL	8664.7	8257.4^b^	95.30	80.16, 113.30

*ANOVA* analysis of variance, *AUC*_*inf*_ area under the concentration–time curve from time 0 to infinity, *AUC*_*last*_ area under the concentration–time curve from time 0 to the time of the last quantifiable concentration, *CI* confidence interval, *C*_*max*_ maximum observed concentration, *LS* least squares, *PK* pharmacokinetic

^a^(Lansoprazole + quizartinib)/(quizartinib alone)

^b^*n* = 31

### Effect of lansoprazole on AC886 and quizartinib + AC886 PK

Geometric LSMs for AC886 *C*_max_, AUC_last_, and AUC_inf_ decreased after lansoprazole exposure, by approximately 23%, 25%, and 18%, respectively (Table 4). The PK profiles of AC886 in the two treatment groups were similar, with comparable *T*_max_ (6.0 h with lansoprazole + quizartinib and 5.0 h with quizartinib) and *T*_1/2_ (101.0 h with lansoprazole + quizartinib and 98.3 h with quizartinib). The quizartinib + AC886 geometric LSMs for *C*_max_, AUC_last_, and AUC_inf_ were also lowered by approximately 15%, 8%, and 7%, respectively, after lansoprazole treatment (Table 5). Median *T*_max_ was 4.0 h in both treatment groups.

**Table 4:** Statistical comparisons (ANOVA) of AC886 PK parameters after a single 30-mg dose of quizartinib alone or with lansoprazole

PK parameter	Quizartinib	Lansoprazole + quizartinib	Ratio of geometric LS mean, %^a^	90% CI for ratio of geometric LS mean, %
	Geometric LS mean, *n* = 30	Geometric LS mean, *n* = 32		
*C*_max_, ng/mL	21.2	16.3	76.93	57.13, 103.60
AUC_last_, ng h/mL	2778.7	2088.3	75.15	61.09, 92.45
AUC_inf_, ng h/mL	2847.8^b^	2329.3^b^	81.79	67.50, 99.11
Mean *T*_1/2_ (SD), h	98.3 (26.8)^b^	101.0 (30.6)^b^		
Median *T*_max_ (min, max), h	5.0 (4.0, 72.0)	6.0 (3.0, 72.0)		

*ANOVA* analysis of variance, *AUC*_*inf*_ area under the concentration–time curve from time 0 to infinity, *AUC*_*last*_ area under the concentration–time curve from time 0 to the time of the last quantifiable concentration, *CI* confidence interval, *C*_*max*_ maximum observed concentration, *LS* least squares, *PK*, pharmacokinetic, *SD* standard deviation, *T*_*1/2*_ apparent terminal phase elimination half-life, *T*_*max*_ time to reach maximum plasma concentration

^a^(Lansoprazole + quizartinib)/(quizartinib)

^b^*n* = 29

**Table 5:** Statistical comparisons (ANOVA) of quizartinib + AC886 PK parameters after a single 30-mg dose of quizartinib alone or with lansoprazole

PK parameter	Quizartinib	Lansoprazole + quizartinib	Ratio of geometric LS mean, %^a^	90% CI for ratio of geometric LS mean, %
	Geometric LS mean, *n* = 30	Geometric LS mean, *n* = 32		
*C*_max_, ng/mL	127.3	108.2	84.99	77.42, 93.30
AUC_last_, ng h/mL	11,525.3	10,561.8	91.64	83.84, 100.16
AUC_inf_, ng h/mL	12,012.5^b^	11,127.3^b^	92.63	83.94, 102.22
Median *T*_max_ (min, max), h	4.0 (2.0, 8.0)	4.0 (3.0, 8.0)		

*ANOVA* analysis of variance, *AUC*_*inf*_, area under the concentration–time curve from time 0 to infinity, *AUC*_*last*_ area under the concentration–time curve from time 0 to the time of the last quantifiable concentration, *CI* confidence interval, *C*_*max*_ maximum observed concentration, *LS* least squares, *PK* pharmacokinetic, *T*_*max*_ time to reach maximum plasma concentration

^a^(Lansoprazole + quizartinib)/(quizartinib)

^b^*n* = 29

### Safety

Quizartinib administered with or without lansoprazole was generally safe and well tolerated by healthy subjects. Twenty subjects [31.7%; 9 (30.0%) in the quizartinib-alone group and 11 (33.3%) in the lansoprazole + quizartinib group] experienced at least one treatment-emergent AE (TEAE) following quizartinib treatment on day 5. The most common TEAEs occurring in either treatment group were headache (3 subjects in the quizartinib-alone group, 10%), upper respiratory tract infection [2 (6.7%) and 3 (9.1%) subjects in the quizartinib-alone and lansoprazole + quizartinib groups, respectively], and muscle tightness (2 subjects in the quizartinib-alone group, 6.7%) (Online Resource 2). Of the 20 subjects who experienced a TEAE, the majority (19 subjects; 95%) reported TEAEs of mild severity. One subject in the quizartinib-alone group experienced headache of moderate severity that was considered possibly related to quizartinib. Three subjects experienced at least one quizartinib-related TEAE, including nausea, diarrhea, dizziness, headache, and fatigue. None of these quizartinib-related TEAEs occurred in more than one subject in either treatment group. There were no severe or serious TEAEs in this study.

No significant abnormalities in vital signs, hematology, or clinical chemistry were observed during the study. Most subjects in the quizartinib-alone and lansoprazole + quizartinib groups had a maximum QTcF value of ≤ 450 ms and a maximum change from baseline of ≤ 30 ms. Two (6.7%) and zero subjects in the quizartinib-alone and lansoprazole + quizartinib groups, respectively, had maximum QTcF > 450 ms but ≤ 480 ms. None of the subjects had a maximum QTcF value > 480 ms.

## Discussion

Patients with AML often experience gastrointestinal complications, which may result in concomitant use of ARAs (such as PPIs, antacids, and H2 blockers) as a part of disease management [13]. However, concomitant use of ARAs may compromise the therapeutic benefit of oral TKIs, potentially through altered solubility and bioavailability. Although many factors may influence TKI absorption, one major determinant is pH-dependent solubility [16]. A chemical structure analysis of 39 oral targeted cancer therapies in clinical development identified at least 11 compounds (28%) predicted to have pH-dependent solubility [15]. Of recently approved orally administered cancer therapeutics, > 50% are characterized as having pH-dependent solubility. Clinical experience also indicates drug–drug interaction between PPIs and some TKIs, such as nilotinib or dasatinib [21–23]. In vitro assessment of quizartinib dihydrochloride confirmed the compound’s solubility was pH dependent, with low solubility (< 4.0 × 10^−4^ mg/mL) at physiological pH in the intestine. Thus, it was important to examine potential effects of a PPI (the most potent and long-lasting ARA class) on the bioavailability of the tablet formulation of quizartinib to ensure consistent plasma exposure and clinical efficacy and to inform dosing guidance. The metabolite, AC886, is active with similar pharmacology to quizartinib; thus, the total of parent and metabolite represents the pharmacologically active exposure.

PPIs are considered the most effective gastric ARAs, inhibiting acid secretion by as much as 90% and increasing gastric pH to > 4 [24]. Lansoprazole is believed to be transformed into two active species at the secretory surface of the gastric parietal cell that are not present in the systemic circulation [25]. Although the elimination half-life of lansoprazole is less than 2 h, an inhibitory effect on gastric acid secretion lasts over 24 h. Following administration of lansoprazole 30 mg once daily for 5 days, mean 24-h intragastric pH increased to 4.9 from a baseline of 2.1; thus, lansoprazole 60 mg once daily was chosen for this study. This clinical study of the quizartinib tablet showed that increased gastric pH with lansoprazole had only a modest effect on the rate and extent of quizartinib absorption, with only ~ 15% mean decrease in *C*_max_ of quizartinib + AC886 and similar median *T*_max_ with coadministration of the tablet formulation of quizartinib and lansoprazole in healthy subjects. Variability in *C*_max_ values was slightly higher when lansoprazole was coadministered. Plasma exposure of quizartinib and total exposure (quizartinib + AC886), as assessed by *C*_max_ and AUCs, decreased slightly after repeated lansoprazole administration. In vitro, quizartinib solubility decreases with increase in pH; thus, it is likely that the quizartinib tablet solubility in the human gastrointestinal tract is reduced when lansoprazole is co-administered. In the tablet formulation, 2-hydroxypropy-beta-cyclodextrin was used as a solubility-enhancing agent and could have contributed to minimizing the effect of the acid-reducing agent. The reduced solubility may cause the increased variability in the absorption of quizartinib in the gastrointestinal tract, as reflected in the increased variability in *C*_max_. However, the small decrease in exposure observed was not expected to be clinically relevant.

Metabolite–parent ratios (AC886/quizartinib) for AUC_last_ and AUC_inf_ were similar with and without lansoprazole, with geometric means ranging from 0.267 to 0.334. *T*_1/2_ for quizartinib and AC886 were similar in both treatment groups. Therefore, quizartinib can also be administered with other gastric ARAs such as antacids and H2 blockers without clinical consequence.

Quizartinib administered as a single dose alone or co-administered with lansoprazole was well tolerated in this study. The most common TEAEs that occurred with quizartinib treatment were headache, upper respiratory tract infection, and muscle tightness. Most of these events were mild in severity. Most subjects had maximum QTcF values of ≤ 450 ms and maximum change from baseline ≤ 30 ms, with 2 subjects with maximum QTcF ranging from > 450 to ≤ 480 ms (quizartinib-alone group). However, no subjects had a maximum QTcF value > 480 ms.

In conclusion, although the quizartinib dihydrochloride drug substance has pH-dependent solubility in vitro, the PPI lansoprazole had minimal effect on quizartinib PK. Therefore, quizartinib as a formulated tablet can be administered with or without ARAs such as PPIs, antacids, and H2 blockers.

## Electronic supplementary material

Below is the link to the electronic supplementary material.

Supplementary material 1 (DOCX 27 kb)

Supplementary material 2 (PDF 105 kb)
