# Supplementary material for: A drug–drug interaction study to assess the potential effect of acid-reducing agent, lansoprazole, on quizartinib pharmacokinetics
Source: Cancer Chemother Pharmacol. 2019 Aug 5;84(4):799–807. doi: 10.1007/s00280-019-03915-1 (PMC6768889; doi:10.1007/s00280-019-03915-1)
Supplement: Supplementary file 1 — Supplementary material 1 (DOCX 27 kb) [file 280_2019_3915_MOESM1_ESM.docx]

**ONLINE RESOURCES**

| Online Resource 1. CONSORT Study Flowchart  **Online Resource 2. Treatment-emergent Adverse Events in Patients Receiving Quizartinib With/Without Lansoprazole (Safety Population)** | | | |
| --- | --- | --- | --- |
| **TEAE, n (%)** | **Quizartinib**  **(n = 30)** | **Lansoprazole +  Quizartinib**  **(n = 33)** | **All patients**  **(N = 63)** |
| Upper respiratory tract infection | 2 (6.7) | 3 (9.1) | 5 (7.9) |
| Headache | 3 (10.0) | 0 | 3 (4.8) |
| Constipation | 1 (3.3) | 1 (3.0) | 2 (3.2) |
| Nausea | 1 (3.3) | 1 (3.0) | 2 (3.2) |
| Fatigue | 1 (3.3) | 1 (3.0) | 2 (3.2) |
| Back pain | 1 (3.3) | 1 (3.0) | 2 (3.2) |
| Muscle tightness | 2 (6.7) | 0 | 2 (3.2) |
| Dysmenorrhea^a^ | 1 (3.3) | 1 (3.0) | 2 (3.2) |
| Musculoskeletal stiffness | 1 (3.3) | 0 | 1 (1.6) |
| Diarrhea | 1 (3.3) | 0 | 1 (1.6) |
| Dizziness | 0 | 1 (3.0) | 1 (1.6) |
| Hypoesthesia | 0 | 1 (3.0) | 1 (1.6) |
| Musculoskeletal pain | 1 (3.3) | 0 | 1 (1.6) |
| Vessel puncture site pain | 0 | 1 (3.0) | 1 (1.6) |
| Nasal congestion | 1 (3.3) | 0 | 1 (1.6) |
| Sneezing | 1 (3.3) | 0 | 1 (1.6) |
| Throat irritation | 1 (3.3) | 0 | 1 (1.6) |
| Hypoacusis | 0 | 1 (3.0) | 1 (1.6) |
| Dermatitis | 0 | 1 (3.0) | 1 (1.6) |
| Phlebotomy | 0 | 1 (3.0) | 1 (1.6) |
| Note: TEAEs were events with a start date on or after the administration of quizartinib on day 5. Patients may have had more than 1 TEAE.  ^a^ Following database lock, one of these events was found to be pre-existing, thus reducing the incidence from 2 to 1.  Abbreviation: TEAE, treatment-emergent adverse event. | | | |
