# Supplementary figures and images for: A drug–drug interaction study to assess the potential effect of acid-reducing agent, lansoprazole, on quizartinib pharmacokinetics
Source: Cancer Chemother Pharmacol. 2019 Aug 5;84(4):799–807. doi: 10.1007/s00280-019-03915-1 (PMC6768889; doi:10.1007/s00280-019-03915-1)

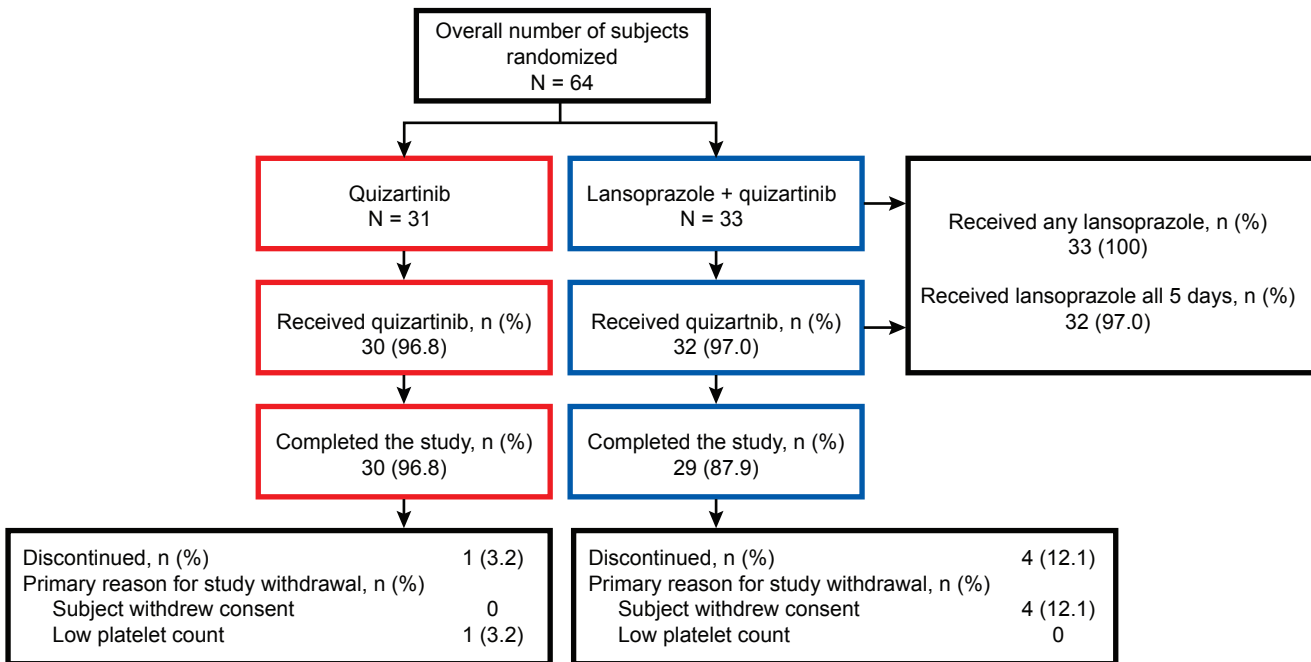

Supplement: Supplementary file 2 — Supplementary material 2 (PDF 105 kb) [file 280_2019_3915_MOESM2_ESM.pdf]
